# A Randomized Controlled Trial of a Care Partner-Assisted Oral Hygiene Intervention for Persons With Mild Dementia

**DOI:** 10.1093/geroni/igaf122.1026

**Published:** 2025-12-31

**Authors:** Bei Wu, Jackie Finik, Catharine Mott, Patricia Poole, Melanie Bunn, Ruth Anderson, Brenda Plassman

**Affiliations:** NYU Shanghai, Shanghai, China (People’s Republic); New York University, New York, New York, United States; Duke University, Durham, North Carolina, United States; The University of North Carolina at Chapel Hill Adams School of Dentistry, Chapel Hill, North Carolina, United States; Duke University School of Nursing, Durham, North Carolina, United States; Duke University, Durham, North Carolina, United States; Duke University Medical Center, Durham, North Carolina, United States

## Abstract

Oral hygiene contributes to oral health outcomes and quality of life. This study evaluates a care partner-assisted intervention to improve oral hygiene in community-dwelling older adults with mild dementia. We conducted a three-arm randomized controlled trial involving 60 participants with mild dementia and their care partners. Participants were randomized into three arms: (1) control (educational booklet only), (2) Treatment Group 1 (TG1: smart electronic toothbrush without coaching), and (3) Treatment Group 2 (TG2: smart electronic toothbrush plus personalized coaching). Plaque and gingival inflammation were assessed at baseline, 3 months, and 6 months using linear mixed models. All groups showed significant reductions in mean plaque and gingival inflammation from baseline. For plaque, significant reductions were observed at both 3 [β=-0.10 (95% CI, -0.15, -0.06)] and 6 months [β=-0.07 (95% CI, -0.12, -0.03)]. For gingival inflammation, significant reductions were observed at 3 months [β=-0.12 (95% CI, -0.19, -0.05)] but not at 6 months [β= -0.05 (95% CI, -0.12 to 0.02)]. In terms of absolute reduction at study follow up, TG2 showed the largest reduction from baseline in mean plaque [3 months: -0.19 TG2, -0.07 TG1, -0.06 control; 6 months -0.15 TG2, -0.07 TG1, -0.01 control] and gingival inflammation [3 months: -0.22 TG2, -0.07 TG1, -0.05 control; 6 months -0.16 TG2, -0.10 TG1, +0.11 control]. A care partner-assisted intervention may support oral health improvements in individuals with mild dementia, with potential sustained benefits. Larger studies with longer follow-up are needed to fully evaluate the efficacy of the intervention.

